# Low-cost, open-source, full-stack software and Arduino-based hardware for control of commercially available animal behavior systems

**DOI:** 10.3758/s13428-026-03096-9

**Published:** 2026-06-29

**Authors:** Scott Miller, Jacob C. Slack, Amol P. Yadav

**Affiliations:** 1 Department of Biomedical Engineering, University of Michigan, Ann Arbor, MI USA; 2 Lampe Joint Department of Biomedical Engineering, University of North Carolina at Chapel Hill and North Carolina State University, Chapel Hill, NC USA; 3 Neurosurgery, School of Medicine, University of North Carolina at Chapel Hill, Chapel Hill, NC USA; 4 Neuroscience Center, School of Medicine, University of North Carolina at Chapel Hill, Chapel Hill, NC USA

**Keywords:** Open-source, Operant conditioning, Behavioral experiments, Full stack, Arduino

## Abstract

Behavioral neuroscience relies heavily on controlled environments, such as operant chambers or “Skinner boxes,” to characterize relationships between external stimuli and the resulting animal behavior. Increasingly, these methodologies are critical for the development of neural interfaces which seek to provide or restore sensations via electrical stimulation. To conduct behavioral experiments, researchers have commonly trusted commercial systems, like those from Med Associates, Inc. While offering reliability, high costs and limited customizability have motivated a push towards open-source alternatives, which often involve the use of inexpensive microcontrollers, custom printed circuit boards (PCBs), and freely available codebases. However, despite these developments, there is a lack of comprehensive software solutions that can integrate seamlessly with commercial or custom hardware for behavioral experiments. In this study, we developed a full-stack application utilizing Angular and Flask frameworks to conduct two-alternative forced choice (2AFC) tasks controlled by an Arduino which interfaces with Med Associates, Inc. operant chamber equipment via a custom PCB. The system was tested by conducting a simple operant conditioning procedure and a spinal cord stimulation (SCS) sensory detection experiment using a custom microstimulator in rodents. The analyzed data demonstrated appropriate behavioral learning and sensory detection thresholds, in alignment with previous SCS behavioral studies which utilized commercial or single-tier systems for control of operant chambers. This work demonstrates the effective integration of an open-source full-stack application with existing commercial hardware that can provide adaptable and scalable means for conducting behavioral experiments, crucial for advancing neural interface technologies.

## Introduction

Behavioral neuroscience offers the opportunity to study cognitive, motor, sensory, and executive functions in animals, enabling us to decipher the relationship between behavior and neural processing. It often involves the analysis of the relationship between an external stimulus and ensuing visual (Hannah et al., 2019), tactile (Ackerley et al., 2014), auditory (Anikin et al., 2021), or gustatory perception (Fahmy & Whitcroft, 2022), providing insights into how animals interact with and process their surroundings. Novel technologies such as brain–computer interfaces and neuroprosthetics depend on behavioral tasks to evaluate their role in the rehabilitation and restoration of functional deficits (Carmena et al., 2003; Chapin et al., 1999; Wessberg et al., 2000). For instance, behavioral experiments involving the assessment of artificial sensations generated by electrical stimulation of the brain (Armenta Salas et al., 2018), spinal cord (Yadav et al., 2021), or peripheral nerves (George et al., 2019) provide an ideal avenue through which to measure whether neural stimulation can restore lost perceptual ability. As such, the methodology for collecting behavioral data in these contexts is crucial for developing effective neural interfaces.

A common approach for conducting behavioral studies in animal models is the use of operant conditioning chambers, or “Skinner boxes,” which are controlled environments that allow researchers to design and employ experimental procedures such as reinforcement scheduling (Fernández-Lamo et al., 2016), go/no-go paradigms (Smith et al., 2023), or two-alternative forced choice (2AFC) tasks (Soma et al., 2014). Companies like Lafayette Instruments (Lafayette, IN, USA), TSE Systems (Berlin, Germany), and Med Associates, Inc. (Fairfax, VT, USA) offer a wide range of commercially available products including complete operant systems, individual components, and software packages. Although companies like Med Associates supply modular components, which allow researchers to develop different configurations for a variety of experiments, these systems are both expensive and limited in use despite the modular design (Lay et al., 2020). The exorbitant costs owing to proprietary technology are due to the assembly and maintenance expense, which can limit or outright prevent research projects from occurring (O’Leary et al., 2018). With the cost of electronic components decreasing over time and a rise in popularity of open-source research systems (Isik & Unal, 2023; White et al., 2019), more opportunities are now available for individuals to design custom systems to accomplish any task. Electronics companies such as Arduino (Monza, Italy) produce open-source microcontrollers with a diverse and inexpensive product line. For instance, the Arduino Uno Rev3 is the most popular microcontroller, used for its versatility and compatibility with different sensors (Junior et al., 2014). It was recently assessed for use in the control of a neuropsychological experiment, and it was found to exhibit stable transistor–transistor logic (TTL) and could be advantageous in the use of wearable experimental devices that are portable, easy to replicate, and reliable (D’Ausilio, 2012).

The design of alternative inexpensive systems has been explored by many research groups, who have combined different technologies to perform behavioral experiments. While the hardware used by each group remained nearly constant, using Arduino and a custom-designed circuit, the software utilized to execute protocol and provide a user interface (UI) has varied. Some groups purchased software packages from established companies to interface with custom hardware (Chandra et al., 2022; Rizzi et al., 2016; Steurer et al., 2012), while others developed unique Arduino scripts (Devarakonda et al., 2016), Pygame programs (O’Leary et al., 2018), and software packages (Kapanaiah et al., 2021) which commonly utilize single-tier architecture where UI, logic, and data handling are contained in a single environment.

Despite the presence of numerous alternative systems, there has been little use of comprehensive and readily scalable systems, such as custom client-server full-stack applications, that can seamlessly interface with existing commercial hardware. A full-stack application consists of three layers: a client application that features a UI for users to interact with, a back-end service that processes logic and commands sent from the client application, and a data layer that stores data used by the other two layers. The back-end service can connect to one or multiple client applications. A full-stack application can be made from established stacks (LAMP, MEAN, etc.) or from a combination of established frameworks, such as Angular (Google, Mountain View, CA, USA) and Flask (Cincović et al., 2019). For instance, one group demonstrated the use of a MEAN stack application with a representation state transfer (RESTful) application programming interface (API) to collect ambient temperature data in real time from an ATMEGA328 microcontroller (Ateml, San Jose, CA, USA) and MCP9808 temperature sensor (Microchip Technology, Chandler, AZ, USA) to display to a user and store in a local database (Poulter et al., 2015). Here, we present the development and implementation of a full-stack application using Angular and Flask frameworks integrated with Arduino-based hardware capable of controlling commercially available operant conditioning equipment. This system was then tested for real-time feasibility by conducting two-alternative forced choice (2AFC) tasks and collecting behavioral data with rodents.

## Methods

All animal procedures were performed according to prior approved protocols by the University of North Carolina at Chapel Hill #24.006 and Indiana University #21055 Institutional Animal Care and Use Committees and in accordance with National Institutes of Health Guide for the Care and Use of Laboratory Animals NIH Publication No. 80–23. Seven Long-Evans rats (250–400 g) were used in the testing of multiple behavioral paradigms for validating the system in real time.

### System overview

**Fig. 1:**
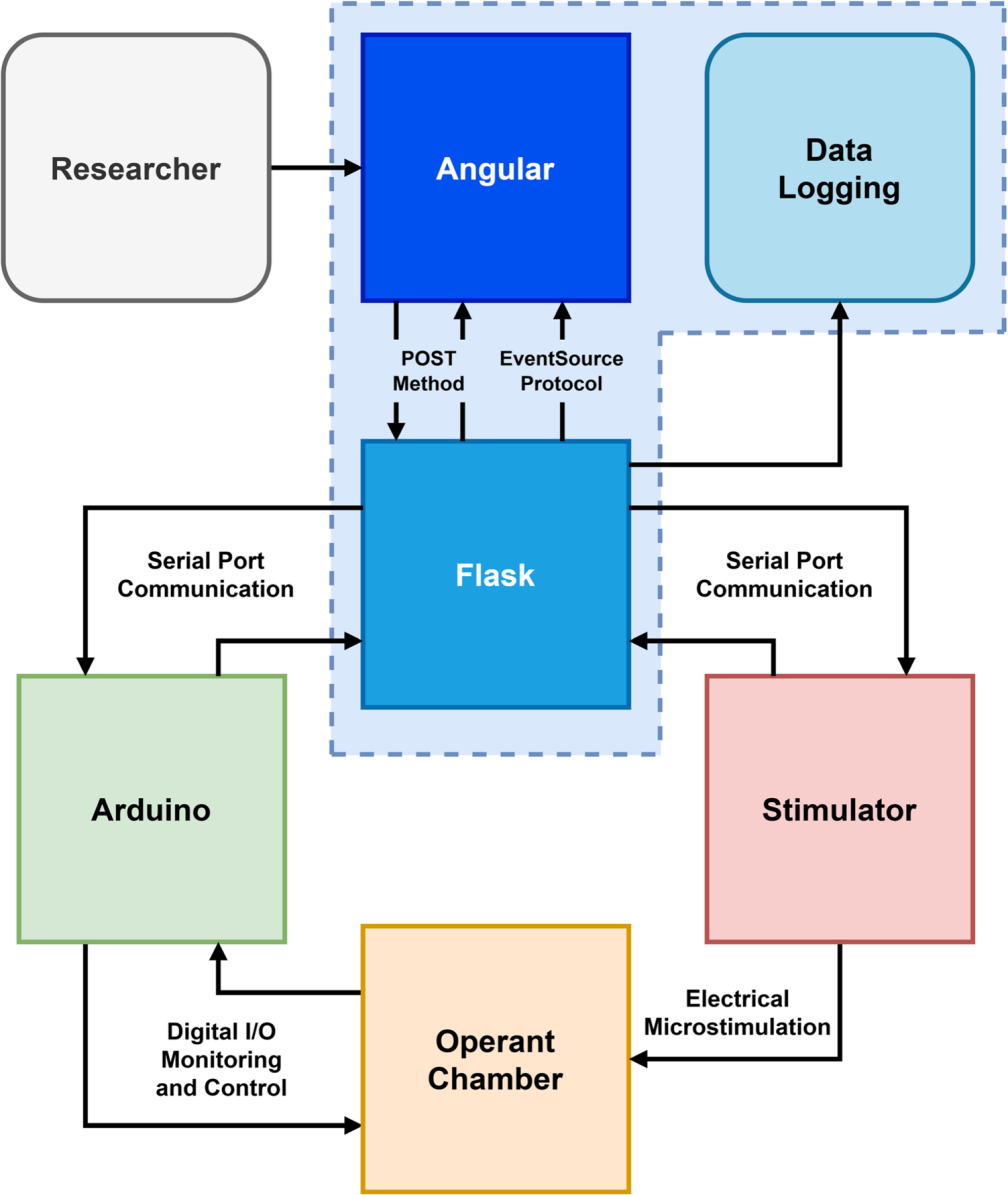
Overview of operant chamber application. *Note.* System components running on a researcher’s computer are outlined in the dashed blue box.

The proposed system uses open-source software, Angular and Flask, to create a full-stack application and publicly available resources to design a printed circuit board (PCB) that allows an Arduino Uno Rev3 to interface with the digital logic of Med Associates, Inc. (VT, USA) products and a custom programmable microstimulator (Hanson et al., 2012). Users have dropdown menus and digital buttons to connect associated hardware and select experimental settings, which are sent via hypertext transfer protocol (HTTP) requests to be processed in the RESTful API. Once the experiment begins, developed programs flashed onto the Arduino and a custom programmable microstimulator communicate via serial ports with the RESTful API to execute the desired experimental protocol. An overview of the interface can be found in Fig. 1. Code, design files, and detailed documentation for the system are available online (GitHub Repository). In the following sections, locations of files within GitHub will be provided when applicable using “…/” to indicate the top level of the repository “open-source-rat-behavior/.” Software prerequisites, installation, and launching instructions can be found in the README at the top level of the repository (…/README.md). Two main versions of the system are available: (1) v1.0, which was used for initial training and spinal cord stimulation detection experiments (detailed in the “Operant conditioning tasks” section below), and (2) v2.0, which was used for initial training and auditory detection experiments with improved back-end functionality, specifically in serial port communication.

### Operant chamber

The operant chamber and components consist of an assortment of electrical/electromechanical peripherals and Med Associates, Inc. products that were inherited from previous experimental protocols (Slack et al., 2024; Yadav et al., 2021). The modular test chamber (ENV-009A, Med Associates, Inc., VT, USA) is contained within a sound-attenuating cubicle (ENV-016M), which is equipped with a SmartCtrl connection panel (DIG-716) that features 16 output channels and eight input channels. Each channel is a three-pin connection to the peripheral devices via mini-Molex extension cables (SG-216A, SG-218). One pin is fixed at +28 V to supply power to the peripheral devices, another pin acts as a ground, and the third pin conducts digital logic. The voltage of a logical high is +28 V, and the voltage for logical low is ground. Peripheral devices connected to the input channels, like the photobeam lickometer (ENV-251L), generate a digital logic level based on the state of the sensor. Peripheral devices connected to the output channels, like solenoid valves for water reward delivery, lighting, and sound indicators, respond to digital logic levels provided by another circuit. Each channel’s digital logic is mapped to a particular pin of a DB25 connector, where a DB25 interface cable (SG-210CB) is used to send and receive data from an external source. In the case of the Med Associates system, the DB25 cable is routed to an interface cabinet connected to a computer where control of reading and writing of peripherals is programmed with proprietary software. For the open-source system, the cable connects to a PCB and is interfaced with an Arduino microcontroller. Control of eight peripheral devices (2 inputs + 6 outputs) was required (Table 1) to conduct the behavioral experiments detailed in the “Operant conditioning tasks” section. To additionally demonstrate the customizability of the system, seven of the peripherals were interfaced with the SmartCtrl connection panel, while the eighth component, an infrared (IR) break-beam sensor (Adafruit Industries, NY, USA) that operates at a lower voltage than the commercial hardware, was interfaced directly by the microcontroller.

**Table 1:** Operant chamber components controlled by Arduino and interfaced with a Med Associates SmartCtrl connection panel and custom PCB

Peripheral	Functionality	Channel	Quantity
Water reward solenoid valve	Write HIGH to deliver reward and LOW to stop	Output	2×
Door-panel solenoid valve	Write HIGH to open and LOW to close chamber door	Output	2×
Sound indicator	Write HIGH to generate sound and LOW to stop	Output	1×
House light	Write HIGH to turn on and LOW to turn off chamber light	Output	1×
IR motion sensor	Read HIGH nominally and LOW when beam is broken	Input	2× (total)
Photobeam lickometer			1×
Break-beam sensor			1×

### Custom PCB

**Fig. 2:**
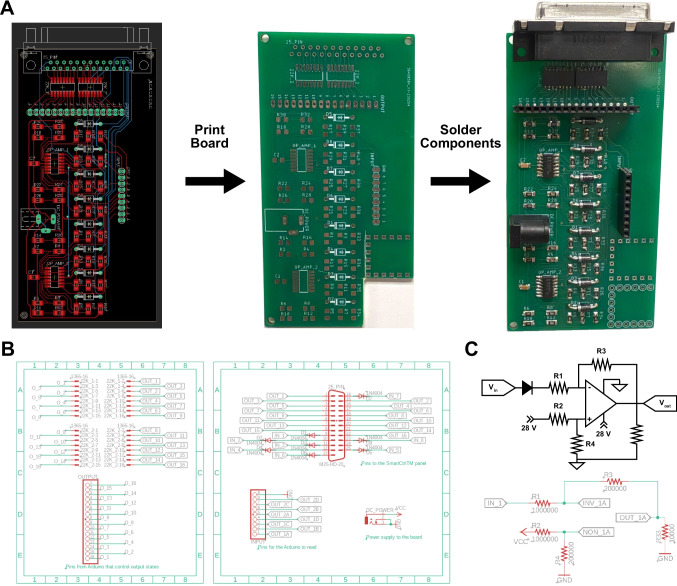
Custom PCB for interfacing Arduino with commercial hardware. *Note.* **A** PCB layout designed in software (left) is printed (middle) and assembled by soldering components to the board (right). **B** Schematic of I/O routing between the SmartCtrl connection panel and Arduino. **C** Diagram (top) and schematic (bottom) of step-down circuit using a differential amplifier configuration attenuates the input voltage (V_in_) to a desired output voltage (V_out_). For a +28 V logic: R1 = R2 = 1 MΩ and R3 = R4 = 200 kΩ. For a +13.69 V logic: R1 = R2 = 1 MΩ, R3 = 300 kΩ, and R4 = 169 kΩ. Schematics in **B** and **C** are used to generate the layout in **A**. The full schematic and board files are available on the GitHub repository (…/Design Files/PCB_schematic.sch, PCB_board.brd)

To enable communication between the Arduino and commercial hardware, a custom PCB was designed in Autodesk EAGLE (San Francisco, CA, USA) and fabricated by JLCPCB (Shenzen, China) (Fig. 2A, left and middle). The PCBs were assembled manually by soldering components purchased from Digikey (Thief River Falls, MN, USA), shown in Table 2 (Fig. 2A, right). Jumper cables were used to connect the PCB to the Arduino, and a DB25 receptacle (Amphenol ICC, Endicott, NY, USA) was used to connect the PCB to the SmartCtrl connection panel. The appropriate input/output (I/O) routing (Fig. 2B) was determined by applying inputs across each of the DB25 receptacle pins and measuring outputs on the SmartCtrl connection panel. A generic AC/DC desktop adapter provides a +28 DC voltage to power the active components of the PCB and the SmartCtrl connection panel to power the operant chamber. A detailed parts list as shown in Table 2 can be found in the GitHub repository (…/docs/*Parts List.xlsx*) along with PCB design files (…/Design Files).

**Table 2:** List of electrical components for the custom PCB interface

Items	Digikey part number	Quantity	Unit cost (USD)	Total cost (USD)
Arduino Uno Rev3	1050-1024-ND	1	27.60	27.60
USB 2.0 cable type A/B	1528-62-ND	1	2.95	2.95
AC/DC desktop adapter 28 V	2034-TRH50A280-01E03VI-ND	1	23.17	23.17
Power cord	EPS614-ND	1	3.91	3.91
DC power jack	1939-G-014-4-ND	1	1.02	1.02
4 Circuit op amp	296-48048-ND	2	2.03	4.06
0.1 µF SMT capacitor	399-C1206C104K5RAC7800CT-ND	2	0.1	0.2
1 MΩ SMT resistor	A129846CT-ND	16	0.1	1.6
200 kΩ SMT resistor	118-CR1206AFX-2003ELFCT-ND	12	0.12	1.44
10 kΩ SMT resistor	A130183CT-ND	8	0.1	0.8
300 kΩ SMT resistor	CRS1206-FX-3003ELFCT-ND	2	0.3	0.6
169 kΩ SMT resistor	311-169KFRCT-ND	2	0.1	0.2
8 22 kΩ isolated resistor network	4816P-1-223LFCT-ND	2	1.53	3.06
1N4007 through-hole diode	1N4007-TPMSCT-ND	8	0.22	1.76
DB25 female socket	609-5879-ND	1	1.49	1.49
DB25 cable assembly	2768-M01-017-ND	1	12.95	12.95
20 pk jumper wire M/M	1568-1512-ND	2	2.1	4.2
9 Position receptacle	2057-2RS1-09-G-ND	1	0.79	0.79
8 Position receptacle	2057-2RS1-08-G-ND	1	0.75	0.75
JLC printing and shipping		1	16.24	16.24
Total cost:				108.79

Component costs are listed based on the date of purchase

The digital logic conducted on the input channels from the Med Associates, Inc. products is a logical high of 28 V, which is greater than the rated voltage of the I/O pins on the Arduino microcontroller. Therefore, a step-down circuit that would not disrupt the digital logic was required to interface the two technologies. A differential amplifier using an operational amplifier, shown in Fig. 2C, was designed for this purpose. One input of the differential amplifier is fixed at +28 V, which is also the positive voltage supply of the operational amplifier (Texas Instruments, Dallas, TX, USA). A decoupling capacitor (KEMET, Fort Lauderdale, FL, USA) is placed near the power supply to reduce noise at the terminal. The other input of the differential amplifier (V_in_) is the digital logic from the operant chamber, which is isolated by a diode (Micro Commercial Components, Simi Valley, CA, USA), that can be either +28 V or 0 V. The only exception to this digital logic is the photobeam lickometer, which instead has a voltage of 13.69 V for the logical high state. Resistor values to attenuate the operant chamber’s digital logic were carefully selected and are indicated in the Fig. 2C caption. A consequence of this design is that the differential amplifier acts as a digital inverter, which does not affect the performance of the circuit but is an important consideration in interpreting the digital state of the input channels.

The voltage required to influence the digital logic of the output channels of the Med Associates, Inc. products is 28 V, which the Arduino cannot supply. Therefore, a step-up circuit that would not disrupt the digital logic was required to interface the two technologies. It was found that the voltage applied to the output channel was not as important as the applied current, where a current of 134–267 µA could modify the digital logic of the operant chamber. A 22 kΩ resistor (Bourns, Inc., Riverside, CA, USA) was implemented to provide a current within this range.

### Arduino microcontroller

An Arduino Uno Rev3 featuring 14 digital I/O pins was used to interface with the SmartCtrl connection panel. As the SmartCtrl connection panel has a total of 24 digital I/O pins, an Arduino Uno does not have the capability to control all I\O channels. However, this only becomes an issue when designing experiments with more components or components requiring multiple channels for operation. In such cases, an Arduino Mega 2560 Rev3 which features 54 digital I/O pins can be utilized. As mentioned previously, only eight channels are required for complete operation of the operant chamber used here. Source code for the microcontroller programs is available in the GitHub repository (…/Arduino Files/).

Programing of the microcontroller was accomplished using the Arduino integrated development environment (IDE), which aims to control and monitor peripheral components, provide bidirectional communication with the RESTful API, and run operant conditioning experiments. Writing and reading of digital pins on the Arduino, connected directly or through the I\O channels on the SmartCtrl connection panel, provide functionality for the components. Serial port communication allows the microcontroller to send and receive information to/from the RESTful API. Information, such as the state of a pin or the time since device startup, is sent from the Arduino by printing to the serial port, which can be read in Flask, logged, and displayed in the user interface (UI). Similarly, a user can send commands from the UI which include manual control of components or updating parameters of an experiment. These commands are read by the Arduino and then processed accordingly to update a variable or read/write a digital pin. The specific structure of the program for running operant conditioning experiments is detailed in the “Operant conditioning tasks” section.

### Programmable microstimulator

Investigation of sensory thresholds involves the presentation of a selected stimulus. In the experiment presented here, electrical stimulation of the spinal cord was chosen based on previous experience studying spinal cord stimulation (SCS) (Slack et al., 2024; Yadav et al., 2021). To do this, a custom, four-channel, current-controlled microstimulator was used to deliver cathodal-leading, bipolar, biphasic patterns (Hanson et al., 2012). Stimulation was delivered in a rat implanted with flat two-contact platinum electrodes located epidurally under the T3 vertebra. SCS pulse-trains were aperiodic, with intervals between pulses generated by a gamma distribution using a coefficient of variation of 0.8 at a mean interval of 50 Hz. SCS was delivered for 2 s, or 100 pulses, with amplitude ranging from 25 to 250 µA, pulse width of 200 µs, and inter-phase interval of 50 µs. The stimulator features a Tiva C Series LaunchPad EK-TM4C123GL (Texas Instruments, Dallas, TX, USA), which houses an ARM Cortex-M4F CPU. The open-source Energia IDE, similar to the Arduino IDE, was used to program stimulator functionality. As with the Arduino, reading and writing to the serial port allowed for communication with a computer where commands could be given to change stimulation parameters, such as frequency and amplitude, and to deliver stimulation in real time.

### Angular framework

**Fig. 3:**
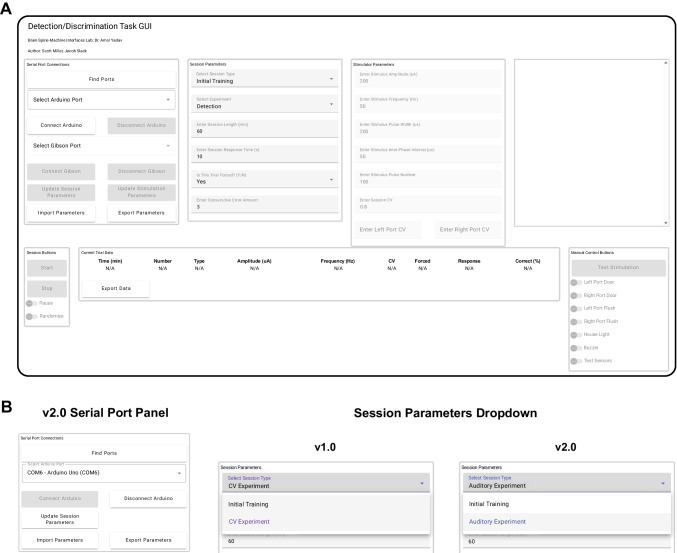
Application GUI for controlling operant chamber components and conducting 2AFC experiments. *Note*. **A** GUI layout for Version V1.0. **B** Example differences in GUI components between v1.0 and v2.0 serial port panel (left) and Session Parameters dropdown (right). Additional screenshots of the GUI with differences between software versions and example Flask and Angular terminal outputs are available on the GitHub repository (…docs/GUI Screenshots with Terminal Outputs.png)

The client application is based on Angular, with NodeJS (OpenJS Foundation, San Francisco, CA, USA) and Git (GitHub, San Francisco, CA, USA) installed to support the framework. Source code is available on the GitHub repository (…/Angular Files/; v1.0, v2.0). The application files were edited in Visual Studio (Microsoft, Redmond, WA, USA). The Angular CLI tool is optional for creating and executing Angular applications but was installed and used for the convenient commands it supplies. Angular Material is a library utilized for UI components. This application was constructed as a single-page application (SPA) that provides a graphical user interface (GUI) for users to manipulate the operant chamber through buttons on the screen (Fig. 3). The application can be executed from the PowerShell located within Visual Studio 2022, the command terminal using appropriate Angular CLI commands, or a batch script which launches both the front end and back end (*start-full.bat* and *start-full-conda.bat* available at the top level of the GitHub repository). Two service classes were developed within the application to carry out HTTP (flaskService) and BehaviorSubject (sseService) protocol between the client-end application and RESTful API, which are used by both components (device-startup and run-trial) present within the application. A detailed sequence diagram of component interactions and screenshots of the launched GUI are available on the GitHub repository (…/docs/FullStackSequenceDiagram.png, GUI Screenshots with Terminal Outputs.png).

Necessary services that the client application performs were organized into two service classes. The first service class (flaskService) is responsible for guiding all POST requests that the client application sends to the RESTful API, alongside the responses returned once the service is completed. Several functions are defined to manage unique categories of UI components (i.e., selection menus, buttons) to set appropriate key–value pairs in an HTTP parameter for the service to process. The second service (sseService) class establishes a BehaviorSubject observable that identifies the server sent events (SSE) data stream as an event source, which emits the last value emitted by the event source, once either the Arduino or programmable microstimulator connect. Additional handlers are present to indicate successful creation of the BehaviorSubject and to reconnect the observable if an error occurs.

The HTML and functionality of the client application were organized into two components that use the service classes through dependency injections. The first component (device-startup) includes selection menus, form fields, and buttons that are pertinent in setting up the experiment parameters and a view port to display SSE data (Fig. 3A, top four panels). In version v1.0, the Arduino and programmable microstimulator each have their own selection menu to display the filtered list of communication (COM) ports that the device is connected to and two buttons to connect and disconnect the device (Fig. 3A, top left menu panel). One button is shared between the devices to search for available ports. Two groups of form fields and selection menus are responsible for letting the user determine the parameters that the session and stimulator will use for the experiment, with each group having its own button to update its respective parameters to the connected devices via the RESTful API (Fig. 3A, middle two panels). In version v2.0, all parameters are routed to a single serial port, as the Arduino is used to both control the task and deliver the stimulus (Fig. 3B, left). Dropdown options also differ based on the programmed experiment (Fig. 3B, right). A view port is present to display the data returned by the RESTful API to the data stream to inform the user with relevant messages and information (Fig. 3A, top right panel). Additional information about the launched server can also be viewed in a separate command terminal window running alongside the GUI. Screenshots of example terminal outputs are available in the GitHub repository (…/docs/GUI Screenshots with Terminal Outputs.png). The second component (run-trial) includes a table displaying data from the current trial and buttons to drive session events, such as eliciting manual control of the hardware components to calibrate and test, exporting session data, or controlling whether the experiment proceeds (Fig. 3A, bottom menu panels).

## Flask micro-framework

The RESTful API is based on Python using Flask for the lightweight framework. Files and dependencies were created within a virtual environment on the local hard drive, and Python files were edited in the lightweight Geany IDE (Cologne, Germany), Integrated Development and Learning Environment (IDLE) IDE (Python Software Foundation, St. Louis, MO, USA), and Visual Studio Code (Microsoft, Redmond, WA, USA); however, the application itself was executed through a command terminal using appropriate Flask commands or via a batch script. Source code for the back end is available on the GitHub repository (…/Flask Project Files/pythonBackend/). RESTful APIs are designed to have a client-server architecture to completely isolate the client end from the back end to improve scalability, statelessness to receive all necessary information from client requests, caching, and a uniform interface to standardize interactions (Fielding, 2000). RESTful APIs are typically equipped with all four HTTP protocols: GET, POST, PUT, and DELETE (Poulter et al., 2015). However, the created back-end service only implemented POST, as the other HTTP verbs were not necessary at this time. The Python file *application.py* is the main file responsible for defining and running the Flask app.

The structure of the Flask RESTful API was defined with four view functions, each provided with their own decorator and uniform resource locator (URL) rule, to organize services designed to mediate interactions between the client application, the Arduino Uno Rev3, and the programmable microstimulator. As mentioned in the previous section, a sequence diagram is available on the GitHub repository (…/docs/FullStackSequenceDiagram.png). The first view function serves as a simple welcome page to visually indicate that the service is running locally within a web browser and provides no other function. The next view function features services that manage serial port objects for the respective hardware and methods to modify experimental parameters. Implemented services include returning to the client application a filtered list of communication (COM) ports that either device is connected to, using *pySerial* to open and close serial port objects and to relay new parameters from the client application to either device once the serial port object has been opened. The next view function relays commands received from button presses on the client application to the serial port object of the respective device, which are encoded in UTF-8 for the Arduino's serial port and converted to bytes for the programmable microstimulator’s serial port. The final view function is responsible for the genesis and maintenance of SSE that is sent to a URL that other sources can observe. A Flask Response object is established with a MIME (Multipurpose Internet Mail Extensions) type of “event stream” and calls a function with a while loop that indefinitely sends data to the event stream when it becomes available. Additionally, HTTP events and serial port communication information are displayed in separate command terminal windows, which run alongside the GUI (screenshots available on GitHub: …/docs/GUI Screenshots with Terminal Outputs.png).

The data yielded originate from the Arduino Uno Rev3 and/or the programmable microstimulator via the opened serial port objects as strings printed to the serial port, which are packaged into a dictionary that is serialized into a JSON string. Try-catch statements are used in these services for error handling. In v1.0, two additional Python files, *serial_functions.py* and *helper_functions.py,* contain functions for handling serial port communication and data exporting, respectively. *The serial_functions.py* can be edited to provide specific or new functionality for reading and writing from the serial port. As an example, if a new component such as a lever is added to the operant chamber, a proper communication method can be written here. In this version, handling of incoming data from the Arduino or microstimulator is generally performed using while loops, with some logic occurring within *application.py*. Because *pySerial* does not have built-in callback functionality (i.e., when *x* data are available on the serial port, call function *y*), while loops using the *in_waiting* method are employed to wait for data to become available for reading. Though functional, unexpected behavior could arise due to instances of blocking while other Flask processes are running, version v2.0 improves upon this by integrating *pySerial* with Python’s built-in *threading* functionality and isolating serial port handling to a custom *ArduinoManager* class. The *serial_thread_functions.py* file defines the *ArduinoManager* class, which enables a serial port “listener” thread that handles incoming data. While not truly parallel, this method provides a non-blocking approach sufficient for these purposes. Additionally, *experiment_handlers.py* contains variables and functions for specific behavioral experiments that can be passed to the *ArduinoManager,* providing improved means for the design of new experiments*.*

## Operant conditioning tasks

**Fig. 4:**
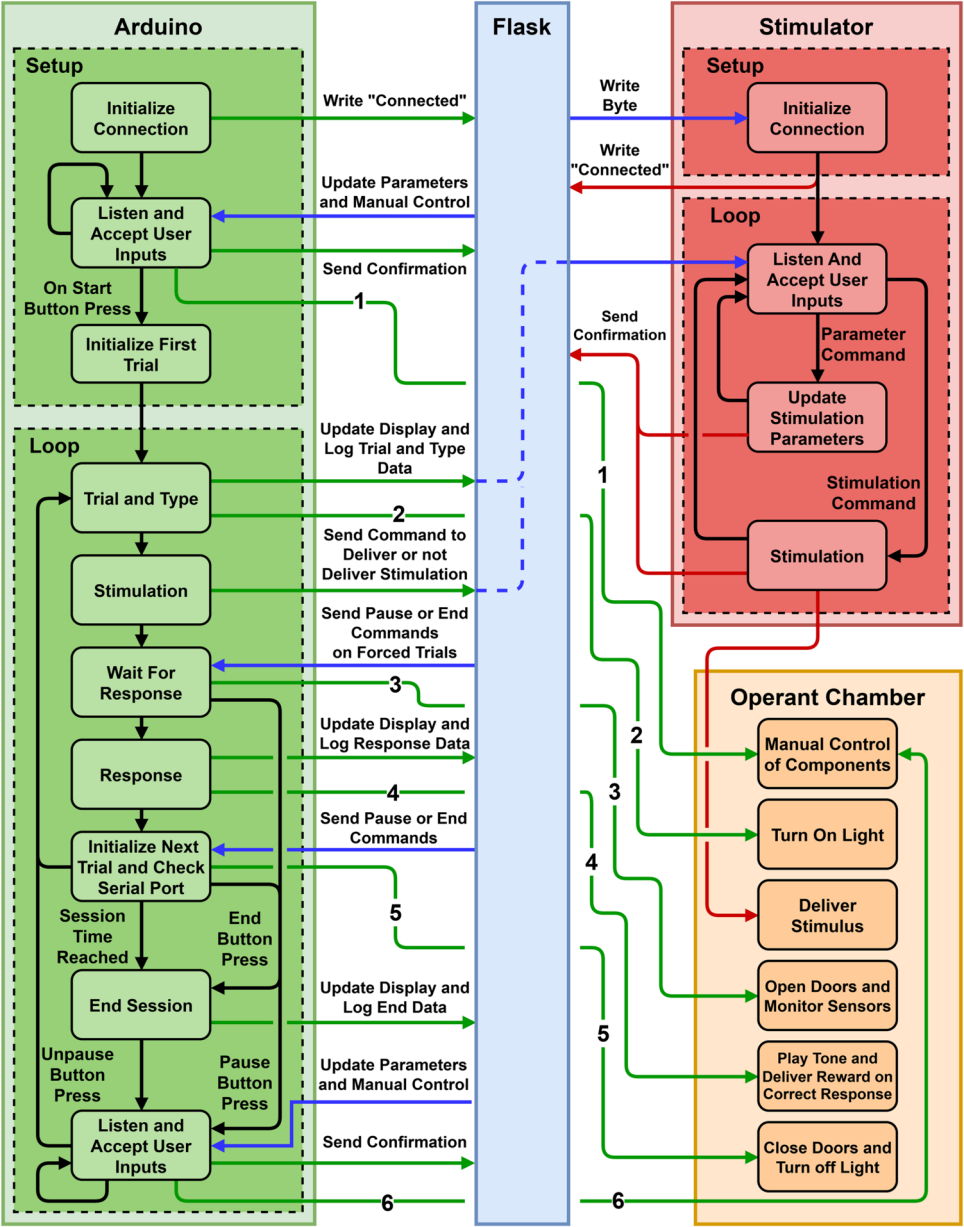
Operant conditioning task flow and device interactions. *Note*. An Arduino microcontroller is programmed following standard *setup()* and *loop()* structure (left, green box) to control an operant conditioning chamber (bottom right, orange). Numbered green arrows indicate direct connections from the Arduino to the operant conditioning chamber. Once a serial port connection is established within the *setup()* function, commands can be sent via Flask through GUI interaction (middle, blue box) to update parameters and control peripherals. Starting an experiment exits *setup()* and enters the *loop()* function, looping through each trial and sending behavioral data via the serial port, which is read by Flask and then updated in the GUI. While an experiment is ongoing, the GUI provides pause and stop functionality. Pausing the experiment enables manual control of the operant box and parameter updating. A programmable stimulator can additionally be controlled via the Flask GUI to enable SCS sensory experiments (top right, red). During the SCS detection task, the Arduino sends a stimulation command to Flask, which relays the command to the stimulator, indicated by the dashed blue lines

To test the efficacy of the system for real-time applications, three experiments following 2AFC task methodology were developed to collect behavioral data from moderately water-deprived rats over a number of sessions. Rats were placed in a Med Associates operant chamber outfitted with the components listed in Table 1; an overview of the task control flow can be seen in Fig. 4. A session consists of a series of multiple trials controlled by the Arduino. Before a session begins, a researcher can update various parameters as shown in the *Session Parameters* grid column in Fig. 3. Additionally, each component in the operant chamber can be manually controlled through the *Manual Control Buttons* grid column in the Angular GUI for testing and verifying proper functionality before starting a session.

The session begins when the *Start* button in the GUI is pressed. Importantly, once the *Start* button is pressed, commands for updating parameters and manual control of components are disabled unless the *Pause* button is pressed, which pauses the session. Commands are then enabled until the *Pause* button is pressed again, unpausing the session. The session will continue until one of two conditions is met: (1) the session time setting is exceeded, or (2) the *Stop* button is pressed in the GUI. A trial begins with the house light turning on and information about the type (left port or right port) of the trial sent to the computer. A 2-s delay occurs followed by the opening of both doors that cover each port. The rat must then choose between the left and right port to receive a water reward within the duration set by the response time parameter. On correct choices, the rat receives a water reward, and a short tone is played through the sound indicator. For incorrect choices or if no choice (no-response) is made within the response time, no water is delivered and a long tone is played. Both doors are then closed, the house light turned off, and the next trial initiated. If forced trials are enabled, when a rat incorrectly chooses a port, the next trial will be a repeat of the previous trial. If the rat consecutively chooses an incorrect port for the number of times set by the consecutive error parameter, only the door corresponding to the correct port will open for the next trial.

In the first experiment, rats (*n* = 5) were trained to alternate between the left and right reward ports, without the presentation of a stimulus, starting with the left port. On choosing the left port, the rat received a water reward. For the following trial, the rat had to select the right port to receive a reward, repeating this pattern until the criteria were met to end the session. The response time was set to 10 s and forced trials were enabled with a consecutive error of 3. The alternating-ports experiment is largely used for habituation and training animals to understand how water can be rewarded. Data collected during this experiment and the following experiments were saved to spreadsheet files and analyzed in MATLAB.

In the second experiment, consisting of an SCS detection task, during the 2-s delay before the doors are opened, a 2-s SCS pulse-train is either delivered or not. If SCS occurs, the rat must choose the left port to receive a water reward. If SCS does not occur, the right port must be chosen. Left and right trials are randomized by the Arduino, and if the researcher presses the *Randomize* switch in the GUI, the amplitude of SCS is randomized in Flask. A total of 10 amplitudes were presented, from 25 to 250 µA, in steps of 25 µA. Toggling off the *Randomized* switch returns the amplitude to the amplitude determined at the beginning of the session. Sessions were conducted until at least 20 trials of each amplitude occurred. A sigmoid function was used to fit the data for the detection task in order to determine the amplitude corresponding to the sensory threshold (where fraction detected crosses 75%). The Arduino code for running the first two experiments can be found on GitHub (…/Arduino Files/operant_task_control/).

For the third experiment, SCS was replaced with an auditory stimulus to demonstrate the configurability of the system for testing other sensory modalities. Rats (*n* =3 ) were trained on an auditory detection task where a fixed suprathreshold 1-s sound cue (3.5 kHz at ~95 dB, XL-2835-TF-LW150-R; PUI Audio, Inc., OH, USA) was paired with the left port and no tone with the right port. Unlike the previous task design, no additional tone was played to indicate correct or incorrect responses. Animals were considered trained when they reached 80% or greater correct responses for three consecutive sessions. For each session and rat, the fraction of correct responses was calculated as the sum of true positives (stimulus present and left port response) and true negatives (stimulus not present and right port response) divided by the total number of completed trials. Forced trials and no-response trials were excluded. For averaging across animals, learning curves were aligned such that session 0 indicated the first session in which the rat satisfied the training criteria. The code for this task is available on GitHub (…/Arduino Files/operant_task_auditory_duration/).

## Results

**Fig. 5:**
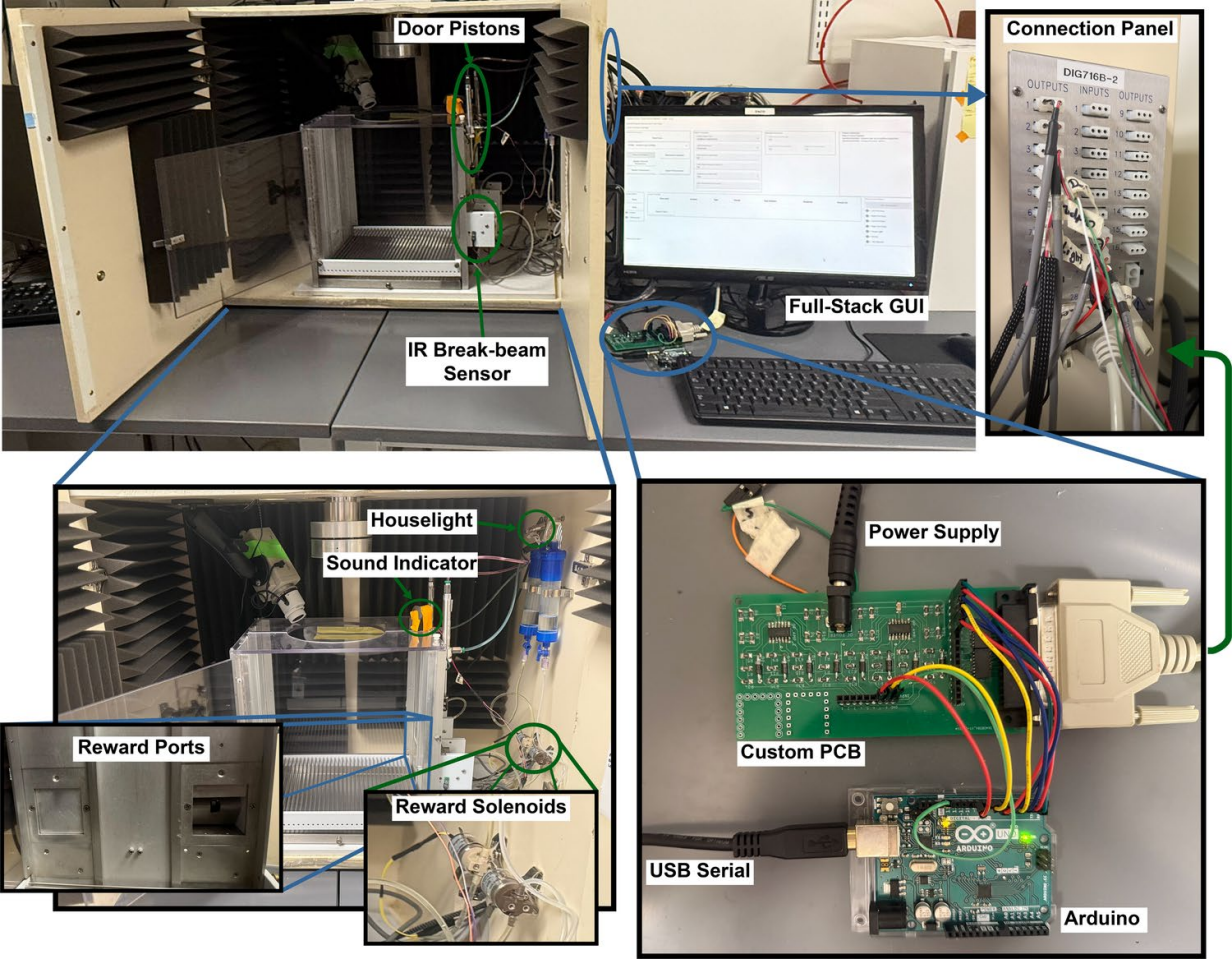
Fully assembled operant conditioning system. *Note*. (Top left) Comprehensive view of assembled system consisting of a proprietary operant conditioning chamber and peripherals, a microcontroller, and computer running the full-stack application. (Bottom left) Focused view of the operant conditioning chamber with labeled peripherals. (Top right) Focused view of the proprietary connection panel for powering and routing I/O control of peripherals. (Bottom right) Focused view of the Arduino microcontroller and custom PCB, which interfaces with the proprietary connection panel

The proposed hardware was demonstrated to successfully interface the Arduino Uno Rev3 with the Med Associates product line using a custom application. One configuration of the step-down circuitry shifted the logic level from a logical high of 28 V to a logical high of approximately 4.67 V and a logical low of approximately 0.31 V, while the other configuration shifted the logic level from a logical high of 13.69 V to a logical high of approximately 4.06 V and a logical low of approximately 1.28 V. Despite the step-down circuitry acting as a digital inverter, both shifted logic levels provided voltage ranges within which the Arduino Uno Rev3 operates to prevent damage. The step-up circuitry successfully utilizes a current below the maximum current rating of the Arduino Uno Rev3 digital I/O pins that can still modulate the digital logic of the Med Associates, Inc. products. The full-stack application can successfully execute experimental protocol, inform the user of real-time data, and save data for later processing. Additionally, it features a simple GUI for users to elicit control and functioning processes like SSE and BehaviorSubject observables to accomplish its designed objective. Integration of the custom PCB and full-stack application with an existing Med Associates behavioral chamber and components can be seen in Fig. 5.

**Fig. 6:**
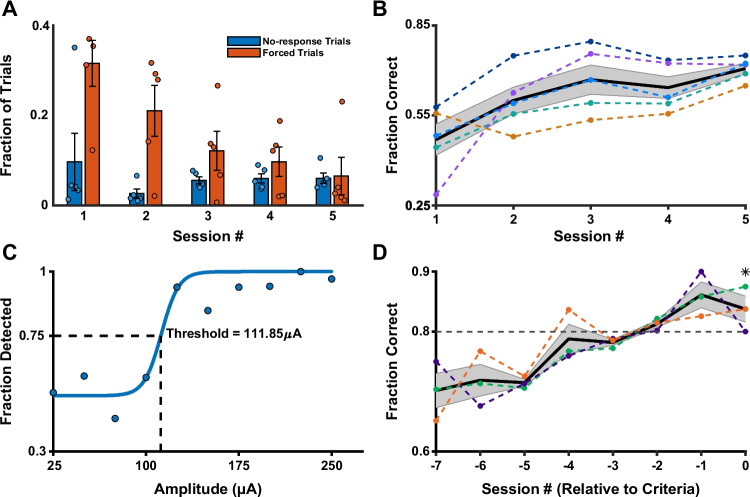
Behavioral data collected across multiple sessions of rats performing 2AFC tasks. *Note*. **A** Bar plot displaying the average number of forced trials and no-response trials for rats (n = 5) performing an alternating-ports 2AFC task. Each day, rats performed a single session (30–60 min). Points represent individual rats and error bars indicate the standard error of the mean (SEM). **B** Fraction of correct responses across session days for the alternating-ports task averaged across rats from **A**. Solid black line and shaded area represent mean ± SEM. Dashed colored lines with points indicate individual rat’s performance. **C** Fraction detected results collected across four sessions of a rat performing an SCS detection 2AFC task. Blue line indicates sigmoid fit of data. Blue dots indicate the fraction of correct responses at the given delivered SCS amplitude across 4 days of training. The intersection of the horizontal dashed line at 75% and the fitted sigmoid indicates the experimentally determined amplitude at sensory threshold. **D** Learning curves for rats (*n* = 3) performing an auditory detection task. Solid black line indicates the fraction of correct responses averaged across rats over the last eight sessions prior to reaching the training criteria. Shaded area represents SEM, and dashed colored lines with points represent individual rat’s performance. Asterisk indicates the session in which criteria were met (≥ 80% for three consecutive days)

The system was then tested for real-time application by running behavioral experiments and collecting data. In the alternating-ports task, the fraction of forced trials decreased and trials where rats did not respond decreased or remained the same across the 5 days of training (Fig. 6A). A reduction in forced trials indicates that consecutive incorrect choices decreased, and a reduction or minimal change in no-response trials indicates that the rat remained consistently motivated across sessions. Additionally, the fraction of correct responses increased across session days (Fig. 6B). These trends demonstrate that rats acclimate to the behavioral system through learning of the alternating-ports task.

For the SCS detection task, data were collected from a rat previously trained on operant condition tasks and implanted with SCS electrodes. The amplitude of SCS was randomly chosen from a range of 25–250 µA, with all other stimulation parameters held constant. Data were collected across 4 days during which the rat performed the task for 30–60 min each day. It was found that as the amplitude decreased, accuracy decreased to chance (50%). Fitting the data to a sigmoid curve revealed a sensory threshold of 111.85 µA (Fig. 6C). Finally, to assess generalizability to other sensory modalities, the system was adapted to conduct an auditory detection task. Following acclimation to the behavioral chamber via the alternating-ports task, rats were trained to respond to a suprathreshold sound cue. All rats achieved better than 80% correct responses for three consecutive days, demonstrating the broader applicability of the system (Fig. 6D).

## Discussion

This work describes an Arduino-based system controlled by a GUI built within the Angular framework and a RESTful API developed within the Flask microframework that is capable of interfacing with Med Associates, Inc. products to perform rodent behavioral tasks. The open-source system was developed as an inexpensive alternative to commercially available behavioral neuroscience equipment for researchers with limited funding while providing equal functionality. Analysis of the collected behavioral data demonstrated both learning characteristics (Fig. 6A, B) and sensory detection thresholds (Fig. 6C) comparable to previous studies that implemented identical operant conditioning procedures controlled by single-tier systems or proprietary Med Associates software (Slack et al., 2024; Yadav et al., 2021). Furthermore, the use of Med Associates components within an open-source design is an appealing prospect, as researchers can repurpose abandoned and previously funded equipment for future projects. Online resources for further reading provided on the repository (GitHub Repository) are readily available to guide this modification process.

The proposed system features beneficial design implementations relative to other systems in the field, offering novel solutions worth expanding upon in future iterations. The major advancement presented is the use of a full-stack application that interfaces with the selected hardware, which features two advantages. The first is that all three layers of the full-stack application are completely independent from each other, so changes can be made to any layer without affecting the rest of the application if the interactions between layers remain intact. This improves the ability to update the application as needed, since each layer can be improved for efficiency, appearance, and compilation of user inputs for a better user experience. While not fully appreciated in the proposed system, which is currently equipped to control a single operant chamber, the second advantage is that the architecture of a full-stack application lends itself to a scalable system. By design, each Arduino is intended to have its own URL endpoint to broadcast SSEs onto, so the only changes theoretically necessary would be the definition of a new URL endpoint for each Arduino added to the system. The front-end application should require no modifications, as each instance of the application, on the same or different devices, would be able to send HTML requests to the URL endpoints presented by the back-end application if they are accessible on the local network.

There are many designs that can perform similarly to the proposed system, each with their own advantages and specialization, with each component having many alternative solutions to meet each group’s needs. The design of the custom PCB does not have to feature operational amplifiers as the means to step down the digital logic, as opto-couplers are another means shown to effectively step up and step down digital logic for communication between devices while simultaneously isolating the circuits (Chandra et al., 2022). The choice of Arduino Uno Rev3 for the microcontroller was motivated primarily by its low cost, straightforward pin header layout, and abundance of online resources. Because the firmware relies on standard serial communication and digital I/O functionality, porting code to an alternative board would require little modification. While not exhaustive, the selection of microcontroller can be changed from the Arduino Uno Rev3 to a different technology, such as the MicroPython pyboard (Kapanaiah et al., 2021), Raspberry Pi 2 (Cambridge, UK) microcomputer (O’Leary et al., 2018), ESP32 boards, or modern versions of Arduino microcontrollers. Products from other operant chamber manufacturers can also be used in a similar fashion, although modifications to the proposed system would be necessary, as each company may develop electronics that operate at different voltages, such as Tucker-Davis products operating at 5 V (Chandra et al., 2022).

If a full-stack application is appealing, then options for frameworks are extensive, as one can select standard stacks such as LAMP, MEAN, or Ruby on Rails; otherwise, a plethora of combinations of stacks are available to choose from, with client-end (ReactJS, jQuery, HTML, Angular, etc.), back-end (PHP, Python, Django, Node.JS, etc.), and database (MySQL, MongoDB, etc.) layers (Bhardwaj, 2021; Gurusamy & Mohamed, 2020). Otherwise, one could conceive a system entirely contained within Arduino code, or with a single application that provides front- and back-end functionality for the selected microcontroller. As the Arduino provides digital I/O functionality through the custom PCB and only requires a serial port interface for communication, a full-stack GUI is not required, and a single-tier system can be used instead. As previously mentioned, Slack et al. (2024) implemented such an application which utilized MATLAB App Designer to interface with the Arduino (and programmable microstimulator) to run alternating-ports and SCS detection and discrimination experiments. However, regardless of the customization or modifications made to the proposed system, the desired design must ensure that appropriate microcontroller operating voltages and software communication protocols are used.

Despite the strengths of the proposed system, there are many improvements that could optimize the system or enable its more widespread use. For example, the custom PCB could be designed to protect against electromagnetic interference (EMI) or electrostatic discharge and safeguard the hardware and users; the code could be updated to host the back-end application on a server to take advantage of the scalable nature of this architecture; the security could be improved to prevent unauthorized users from accessing web applications that might jeopardize sensitive data from the experimental tasks; and finally methods could be implemented to seamlessly reconnect any layer of the system after an unexpected disconnection or failure. Changes to the GUI could improve user experience and input processing, and selecting different components could further reduce costs (Table 2). However, modifying the proposed system, for instance by adding a new behavioral experiment as demonstrated in version v2.0 in our case, would present certain limitations. It would primarily require the appropriate abandoned equipment and secondarily changes to all levels of the application: the front-end application would need updated fields to interact with the new experiment; the back-end application might require new URL endpoints or services; and if necessary, the Arduino code would need to receive new instructions to orchestrate the protocol through the programmable pins. Version v2.0 provides improvements over v1.0 in this regard, with better isolation and organization of back-end code and scripts. Front-end updates of the current approach remain a challenge, but techniques such as object-oriented programming or clearly defining future project goals can lessen this burden without necessitating complete refactoring of the codebase.

## Data Availability

Design files, user manuals, and documentation are available at the repository (GitHub Repository). Links to specific locations in the repository are referenced throughout the paper when applicable. Behavioral data can be made available upon reasonable request.

## Code availability

Code is available at the repository (GitHub Repository).
